# The Progress of Global Antimicrobial Resistance Governance and Its Implication to China: A Review

**DOI:** 10.3390/antibiotics10111356

**Published:** 2021-11-06

**Authors:** Jia Yin, Yu Wang, Xueran Xu, Yinqi Liu, Lu Yao, Qiang Sun

**Affiliations:** 1 Center for Health Management and Policy Research, School of Public Health, Cheeloo College of Medicine, Shandong University, 44 Wenhuaxi Rd, Jinan 250012, China; yinjia@sdu.edu.cn (J.Y.); xuxueran1994@163.com (X.X.); liuyinqifendou@163.com (Y.L.); 18792140221@163.com (L.Y.); 2 NHC Key Laboratory of Health Economics and Policy Research, Shandong University, 44 Wenhuaxi Rd, Jinan 250012, China; 3 Department of Global Health, School of Public Health, Peking University, 38 Xueyuan Road, Beijing 100191, China; ywang@bjmu.edu.cn

**Keywords:** antimicrobial, resistance, global governance, China

## Abstract

China has great potential for engaging in global actions on antimicrobial resistance (AMR) control. This study aims to summarize the process of global AMR governance and provide relevant policy recommendations on how China could take more initiative in the global AMR governance. We searched for academic articles and official document published or issued before December 2020 in e-journal databases, official websites of major organizations, and the relevant national ministries. This review revealed that global action on AMR control has experienced three stages: (1) The beginning stage (1980s and 1990s) when actions were mainly sponsored by high-income countries and AMR surveillance was focused on hospitals; (2) The rapid development stage (2000–2010) when global AMR governance began to concentrate on joint actions in multi-sectors, and developing countries were gradually involved in global actions; (3) The comprehensive stage (2011 to present) when global actions on AMR have covered various fields in different countries. China’s AMR governance has fallen behind at the beginning but recently began to catch up with the global trend. The central government should take a far-fetched view, act decisively and positively towards the global efforts of addressing AMR to play a more active and greater role on the international stage.

## 1. Background

The decrease of mortality caused by infectious diseases has been considered to be partly due to the development of antibiotics. Thus, they are commonly used—and even overused—to prevent and treat bacterial infections in humans and animals. In recent years, the inappropriate use of antibiotics in hospitals has been reported in many low- and middle-income countries (LMICs) [1,2,3]. Moreover, the general public could easily access antibiotics in private pharmacies without prescription in these countries [4,5]. Globally, the consumption of antibiotics is expected to rise by 67% between 2010 and 2030 [6]. The excessive and inappropriate use of antibiotics has contributed to the surge of antimicrobial resistance (AMR). The World Bank reported that AMR would be the leading threat to human health in the next 30 years [7].

The phenomenon of AMR comprises the resistance of bacteria to medicines used to prevent and treat bacterial infections. These drug-resistant strains may infect humans and animals, causing persistent infections that are more difficult to treat than infections by non-resistant bacteria, resulting in prolonged disease duration, increased mortality, and added treatment costs [8]. For example, the duration of drug-resistant tuberculosis (TB) and the associated treatment cost can reach 3–4 times and 100-fold of that of drug-susceptible TB, respectively [9,10]. As AMR has extremely serious impacts on human health, and environmental and socio-economic development, it has become one of the core issues of global health development and health security. According to the World Bank’s estimates, if the problem of AMR could not be effectively addressed, 10 million people would die of it annually by 2050, the annual GDP would be reduced by 3.8% in areas with high resistance rates, and husbandry production would be decreased by 2.6–7.5% [7,11]. Traditional health issues such as maternal and child health, as well as the prevention of infectious diseases could be well-controlled by the efforts of the health sector. According to the framework of “One Health”, a multi-faceted global strategy of cross-sectoral collaboration and communication to secure the health of humans, animals, and the environment, addressing AMR requires action across all levels of society [12]. In recent years, international organizations, including the United Nations (UN), the World Health Organization (WHO), and political forums such as the Group 20 (G20) and the Group 7 (G7), governments and various civil societies have actively developed policies and implemented actions to promote global AMR governance based on the “One Health” approach.

China is one of the largest producers and consumers of antibiotics in the world. In 2013, a total of 162,000 tons of antibiotics were used in healthcare and agriculture in the country [13]. Examples of seriously irrational antibiotic use in humans and animals were commonly reported, and the detection rates of certain resistant bacteria have increased year by year [13,14]. As a country with the world’s largest population and a significant role in global health, China has received high expectations from international communities in global health management, including addressing AMR. However, compared with the international level of AMR governance, there is a significant lag in the initiation and progress of AMR governance in China and its participation in global AMR governance, such as contributing to policy making, attending important international meetings, and providing experts for the global AMR workgroup.

We conducted this review at the macro-policy level with the aim of summarizing the major steps on combating AMR taken by the international community and China, comparing the progress of AMR governance between the two, providing relevant policy recommendations on how China could take more initiative in the global activities on AMR governance. We searched for academic articles, reports, official documents regarding AMR governance published or issued before December 2020 at the major periodical databases of PubMed, ISI Web of Science, Medline, and official websites of global organizations as well as relevant national ministries and commissions of China. Global and national activities and strategies on AMR governance were collected.

## 2. History of AMR

Since the discovery of penicillin in 1928, new antibiotics were continuously identified and developed. During the 1950s and 1960s, a variety of antibiotics were described, such as erythromycin, vancomycin, methicillin, and gentamicin [15,16,17,18]. In the early 1970s, a total of 52 new antibiotics were developed in five years [19]. These antibiotics had a wide range of applications in both the clinic and animal husbandry. However, hardly any antibiotic with a new chemical structure has appeared on the market since 1984 [20]. In parallel, the problem of AMR gradually became more and more aggravated. Penicillin-resistant *Staphylococcus* infections that first occurred in hospitals were increasingly prevalent in the early 1950s [21,22], and multidrug-resistant *Staphylococcus aureus* has become the main cause of nosocomial infections since the 1980s [23,24,25]. Antibiotic resistant strains spread rapidly around the world with the rapid development of global migration and cross-border trade. A newly discovered resistant strain in Europe would spread to the United States (U.S.) in the course of a few months [26]. Scientists realized that the problem of AMR could no longer be solved by discovering more antibiotics, and began to discuss about tackling AMR on a global scale. Since then, international activities on AMR governance have begun.

## 3. Progress of AMR Governance

By reviewing of the global strategies and actions, we divided the progress of global AMR governance into three major stages.

### 3.1. Stage I: The Initiating Stage

The first stage, also called “the initiating stage”, occurred between 1980s and 1990s. Led by the UN and WHO, the activities of combating AMR began to be implemented globally in the early 1980s. In November 1981, the global assembly on AMR governance held by the WHO Antimicrobial Resistance Working Group marked the beginning of global actions against AMR [27]. In 1989, the WHO developed the WHONET software to surveil AMR [28]. By 1996, 160 microbiology laboratories from 31 countries including China had installed this software [29]. In the 1990s, high-income countries (HLCs) like the U.S. and certain European states established their national AMR monitoring systems [30,31]. At this stage, global actions focused on the surveillance of clinical bacterial resistance and were mainly led by HLCs. However, surveillance was often implemented in hospitals without considering AMR in the community [32]. In this stage, few articles regarding actions on AMR control in the low-and middle-income countries (LMICs) were published.

During the same period, China was initiating reform and opening up to globalization. Various fields started to rapidly develop and the country began to correspond with international standards. In the 1980s, the first law on the administration of medicines for human use was formulated and announced by the national legislature [33]. In the 1990s, China was amongst countries affected by nosocomial infections in hospitals. The Ministry of Health (MoH) announced a regulatory guideline, in which the rational use of antibiotics was included as one of the measures to control nosocomial infections [34]. After the mid-1990s, a series of policies were issued by the Ministry of Agriculture (MoA) to control the use and residue of veterinary drugs [35,36,37]. However, there was no specific strategy or action on AMR governance at this stage, and few articles related to AMR in China were published in international journals (Table 1).

### 3.2. Stage II: The Stage of Rapid Development

The rapid development stage took place from 2000 to 2010. In this stage, the consumption of antibiotics increased dramatically in LMICs [38]. Drug resistance was escalating worldwide, and the emergence of multidrug resistant strains and superbacteria raised the global alarm. In this context, international organizations and the HLCs began to be concerned about antibiotic use in agriculture and the environment, as well as AMR in LMICs. Global strategies implemented to fight AMR changed from monitoring to co-governance by several disciplines. This stage is marked by the issue of the *WHO Global Strategy for Containment of Antimicrobial Resistance* in 2001, which provided an interventional framework to reduce AMR from eight important aspects [39]. The WHO, FAO, and OIE carried out either independent or joint actions to combat global AMR. In 2005, the WHO published the first version of critically important antimicrobial agents for human medicine through discussions with the FAO and OIE [40].

At this stage, China accelerated the pace of integration into the international economic society due to joining the World Trade Organization in 2001. Domestic measures against AMR in the medical and agricultural fields began during this period. The first official document on improving the rational use of antibiotics was issued by the national Medical Products Administration in 2003, which limited the activities of selling antimicrobials without prescription in retail pharmacies [41]. During 2004 and 2008, a series of guidance documents were announced by MoH, which aimed to standardize the clinical use of antibiotics and to control nosocomial infections caused by drug-resistant bacteria [42,43,44,45]. In 2005, two nationwide surveillance systems, namely, the “surveillance network for the use of antibiotics in healthcare institutions” and “China AMR surveillance system”, were established by the government [46]. In 2009, a national AMR surveillance program was also implemented in agriculture field [47]. In the environmental field, the only action was a nationwide inspection of hazardous wastes including antibiotic residue [48]. However, these initiatives still mainly concentrated on the clinical monitoring of AMR and the promotion of rational antibiotics use (Table 2).

### 3.3. Stage III: The Comprehensive Stage

During this stage, which covers the period of 2011 to the writing of this review, the high-level meeting of the UN General Assembly and political forum summits raised the importance of AMR from simply a threat to human health to a severe challenge of economic and social development. To attract more global attention to AMR, NGOs and similar organizations began to undertake activities, such as holding sessions, publishing reports, and supporting programs [49,50]. In 2015, the WHO, FAO, and OIE jointly launched an important strategy—*the Global Action Plan for Antimicrobial Resistance*—based on the “One Health” approach [51]. The plan integrates medical, husbandry, agricultural, environmental, and financial sectors into cooperation, and sets joint targets against AMR across all sectors. In the same year, the WHO established the Global Antimicrobial Resistance Surveillance System (GLASS), which aims to standardize the surveillance of AMR through a global collaborative effort [51].

China has also begun to strengthen its governance against AMR since 2011. From 2011 to 2013, three-year special activities on reducing antibiotic use in secondary and tertiary hospitals were implemented across the country [52,53,54]. In 2012, the national government issued a fierce regulation on the clinical use of antibiotics, which classified antibiotics into non-restricted, restricted, and special antibiotics based on their effectiveness, safety, bacterial resistance, and cost [55]. Only physicians with senior professional titles could prescribe all antibiotics, while junior physicians were only allowed to prescribe non-restricted ones. In 2016, 14 national ministries jointly issued the *National Action Plan for the Prevention of Bacterial Resistance 2016–2020* [56]. China became one of the first countries to develop their own National Action Plan (NAP). Based on the NAP, more actions were taken on improving the application of antibiotics for both human and animal use. In the clinical practice, the level of attention paid to antibiotic use and bacterial resistance in children was raised [57,58]. In the agricultural field, lomefloxacin, olaquindox, and six other antibiotics that could be formerly used for both humans and animals were banned in food animals between 2015 and 2017 [59]. Colistin sulfate has been banned from growth promotion in animals since April 2017 [60]. In 2018, 2019, and 2021, the MoA issued a surveillance plan for each antibiotic resistant zoonotic bacteria to ensure the safety of animal source foods [61,62,63]. However, few policies or actions were proposed in the environmental field. The only policies on reducing antibiotic residues and AMR pathogens in the environment were proposed and suggested for development by the deputies in the National People’s congresses held in 2015 and 2017. China also initiated international cooperation projects through the formulation of NAP, and initially participated in global actions against AMR in this period (Table 3).

## 4. Effectiveness of AMR Governance

After nearly four decades of governance, the global has made some achievements in combating AMR. Compared to 2010, 12 and 8 countries of the 40 countries that provided data reported lower volume of human- and animal-use antibiotic consumption in 2020, with most of them were HICs [64]. During 2006 and 2014, the incidence rates of Methicillin-resistant Staphylococcus aureus (MARS) reduced by 18%, 44%, and 16% in Europe, the U.S. and Canada, respectively [65]. However, the overall demand for antibiotics and the total burden of AMR in the world, especially in the low-income areas, continues to rise. Globally, the consumption of human-use antibiotic increased by 65% between 2000 and 2015, and the consumption of animal-use antibiotics is estimated to increase by 12% during 2017 and 2030 [66]. One-third of studies conducted in Asia identified more than 50% antimicrobial compounds with resistance in aquaculture between 2000 and 2019 [67]. Resistance rates for most of the priority antibiotic-pathogens reported by WHO were higher in the LMICs than in the HICs [64].

China has also made some progress on controlling AMR in the last decade. The rate of prescribing antibiotics decreased from 19.4% in 2010 to 7.7% in 2017 for outpatients, and from 67.3% to 36.8% for inpatients in the surveillance hospitals; likewise, the identify rates of most of the concerning resistant pathogens, such as MRSA, carbapenem-resistant klebsiella pneumoniae, and third-generation cephalosporin-resistant Escherichia coli, etc. have declined [68]. However, similar as in other large countries, huge amounts of antibiotics were used in Chinese agriculture [13,66], and the country has seen industrial livestock and poultry develop rapidly to feed a population of 1.4 billion. In 2020, China used an estimate of 43,024 tonnes of antibiotics for animals [64]. Although the government has issued policies to curb the non-therapeutic administration of more than 150 antimicrobial products to food-producing animals, antibiotics are still extensively applied for animal disease prevention and growth promotion in the last few years [69,70].

## 5. Implications to China

### 5.1. Advantages and Disadvantges of China to Contribute to Global AMR Governance

As one of the largest antibiotic producers and consumers, in addition to domestic AMR governance, China could also make more contributions to the global AMR governance. Compared with the global actions on AMR control, China’s actions are relatively lagging behind at each stage. However, China has taken more actions and begun to catch up with the international pace of AMR governance in recent years. The government has realized the importance of AMR governance. In some important international conferences such as the Hangzhou summit, Chinese health authorities expressed their willingness to contribute to global governance initiatives against AMR. China has a great capacity to contribute to global AMR governance. The Chinese healthcare system, led by the National Health Commission with a top-down management approach, has the advantage of rallying resources on effective governance actions, and has gained important successes. China is one of the countries has the highest burden of TB in the word. In 1990s, China implemented the DOTS strategy to control TB throughout the country. Then, along with international TB control projects, the central government provided technical, financial, and equipment resources to TB control institutions across the country [71,72]. With the support of DOTS, the TB prevalence rate dramatically declined, while the treatment success rates rapidly increased [73]. The successful experiences of China’s TB control were subsequently recommended by the WHO to the other high TB-burden countries. In recent decades, more and more Chinese researchers have been involved in studies on AMR and thus contributed to global AMR control. In 2013, a Chinese research team discovered *mcr-1*, a new and transmissible polymyxin-resistance gene [74]. Based on this finding, multiple countries and international organizations elevated the risk level of polymyxin.

Nonetheless, China still faces a number of challenges in both domestic AMR governance and the level of contribution to global AMR governance. At present, the departmental cooperation led by the Medical and Health Hospital of the National Health and Wellness Committee still solely focuses on the clinical monitoring of AMR. This business division mechanism has made China unable to fully carry out its duties necessary to address the resistance issue, such as public education and awareness, as well as drug research. The current expert committee on AMR lacks expertise in the fields of agriculture, environment and policy making. In addition, there are regional differences in AMR governance. The surveillance of antibiotic use in urban areas, as well as secondary and tertiary level healthcare institutions is efficient; however, it is less adequate in rural areas and primary healthcare institutions [75]. Moreover, China’s data sharing with the international community is limited. By April 2020, a total of 92 countries, territories or areas have joint GLASS that is promoted by the WHO [76]. However, despite being one of the major antibiotics consumers, China has neither joined this system nor proposed plans for future participation, putting the country in a passive situation on the international platform of global AMR governance. There are governance policies in the agricultural sector, but these are still insufficient in terms of action and supervision. The environmental sector is also passive and remains on the stage of coordinating the medical and agricultural sectors.

### 5.2. Implications to China to Contribute to Global AMR Governance

There are several implications for China to contribute to global AMR governance. First, a management department whose rank is higher than ministries should be established by the State Council. Its main responsibilities would be to coordinate cooperation among ministries, organize a comprehensive expert committee, prepare monitoring plans and indicator systems of antibiotic use and drug-resistance in various fields, and represent China in international communications. Second, researches on AMR should be facilitated and cross-border cooperation studies should be further expanded. Civil societies and various partnerships, such as “the Belt and Road” initiative, the Shanghai Cooperation Organization, or the 16 + 1 cooperation between Central and Eastern Europe could be utilized to support the global governance program of AMR. Third, more should be done to promote the disclosure of information; information transparency in the agricultural field should be enhanced. Work can be started by gradually sharing China’s population resistance data with the international community. Forth, high-level comprehensive talents should be cultivated, such as using advanced training or secondment opportunities to nurture diplomatic talents in the health field, transforming professional knowledge into diplomatic language. Some global AMR governance experts have played roles in existing international organizations, and these roles should be fully supported. Finally, pharmaceutical innovation should be encouraged. Pharmaceutical companies should be encouraged to invest more in the research and development of new and alternative drugs. The advantages of Chinese traditional medicine should also be fully exploited.

## 6. Limitations

This study has several limitations in terms of data collection. First, we only searched relevant journal articles in three international periodical databases due to the time constraint. Second, this study focused on studies or policies at the macro level without including studies at the molecular level because there are multiple studies regarding AMR at the molecular level and they provided limited information for this topic. Third, few literatures or documents on environmental aspects were collected and analyzed, thus more studies focusing on AMR in this field are recommended. Finally, it would be necessary to collect interpretations from official points of view.

## 7. Conclusions

The global AMR governance has gradually progressed from focusing on clinical AMR surveillance to joint-actions against AMR across multiple sectors in the last forty years. China is catching up with the progress of international AMR governance, but the long-term strategies to tackle AMR in China are more concentrated on monitoring, especially in the agricultural and environmental fields. To contribute more to global AMR governance, the country should view it from the perspective of safeguarding public health, national health security, and the “One Health” principle, take a far-fetched view, and seize the opportunity to act decisively and positively toward the global efforts of addressing AMR, showing responsibility at an international level.

## Figures and Tables

**Table 1:** Policy and regulation milestones on AMR control in China at stage I.

Year	Policy	Issuer	Highlights
1984	China Pharmaceutical Administration Act	The Central Committee of the Communist Party of China	A national law on pharmaceutical producing, selling, management in hospitals, price, advertising, reserving, supplying and surveillance.
1987	Regulations on Veterinary Drug Administration	The State Council	Regulations on the producing, selling, management in veterinary hospitals, advertising, reserving, supplying and surveillance.
1994	Regulatory Guideline for Nosocomial Infection Control (Trial)	MoH	This guideline contains regulations on responsibilities of health administrations and hospitals, infection surveillance, and measures of infection control.
1994	Maximum Residue Limits for Veterinary drug in Animal Foods (Trial) (updated in 1999)	MoA	The MoA defined the Maximum Residue Limits for different kinds of poultry and livestock.
1997	Regulations on types and usage of veterinary drugs used as feed additives	MoA	Regulations on types, usage and dosage for veterinary drugs.
1999	National action plan for surveillance of residues in Animal and Animal Food and official sampling procedure	MoA	This document specified the Laws and regulations on surveillance of residues, responsibilities of the supervisory authorities, sampling procedure, etc.

MoH: Ministry of Health; MoA: Ministry of Agriculture.

**Table 2:** Policy and regulation milestones on AMR control in China at stage II.

Year	Policy	Issuer	Key Point
2003	A notice on strengthening the supervision of antimicrobials sales in retail pharmacies and promoting rational use of antibiotics	FDA	In the retail pharmacies, the antimicrobials which are Rx should be purchased with prescriptions from a licensed physician.
2004	A guideline on clinical use of antimicrobials42	MoH	To promote the rational use of antimicrobials and regulate the behavior of medical healthcare facilities and health care workers.
2005	A notice on the establishment of clinical use of antimicrobials and bacterial resistance monitoring network	MoH	The national monitoring networks of clinical use of antimicrobials and bacterial resistance will be established.
2006	Prescription Administrative Policy43	MoH	The aims of this policy are to standardize prescription management, improve the quality of prescription, promote rational use of medicines, and to ensure medical safety.
2008	A notice on strengthening the control of nosocomial infection causing by multi-drug resistant bacteria44	MoH	This notice requires the hospitals to prevent and control of nosocomial infection and to prevent and control of nosocomial transmission of drug-resistant bacteria.
2008	A notice on further improving the clinical use of antimicrobials45	MoH	Further strengthening the preventive use of antimicrobials in peripheral surgery and gradually establishing an early warning mechanism for the clinical use of antimicrobials.
2009	Animal Bacterial Resistance Surveillance Program (2009)47	MoA	Strengthening the monitoring of animal-derived bacterial resistance, ensuring the safety of animal-derived food and promoting the healthy development of animal husbandry.
2009	A notice on implementing special inspection on the generation and disposal of hazardous waste such as antibiotic residue48	MEP	To organize nationwide inspection on the generation and disposal of hazardous waste such as antibiotic bacteria residue.

MoH: Ministry of Health (former name of National Health Commission); MoA: Ministry of Agriculture; MEP: Ministry of Ecology and Environment; NHFPC: National Health and Family Planning Commission (former name of National Health Commission).

**Table 3:** Policy and regulation milestones on AMR control in China at stage III.

Year	Policy	Issuer	Key Point
2011–2013	Implementation Plan for Special Rectification on Nationwide Clinical Antibiotic Use (2011, 2012, 2013)	NHFPC	From 2011 to 2013, special activity plans on governance antibiotic use were issued every year, focusing reducing antibiotic use in secondary and tertiary hospitals.
2012	Regulations of administrative measures for clinical application of antibacterial agents	MoH	This regulation specified the responsibilities and legal responsibility of health care institutions, health care workers and health administrative departments on management of clinical application of antibacterial agents.
2015	National five-year action plan on comprehensive management of veterinary drugs (antimicrobials) (2015–2019)	MoA	In this plan, the government propose the five-year targets of zero use of veterinary drug prohibited, >97% qualified rate of veterinary antimicrobials, >97% qualified rate of surveillance on residue of veterinary drugs.
2016	National Action Plan on antimicrobial resistance (2016–2020)	NHFPC	To implement comprehensive management strategies at the national level, and strengthen publicity and education and international exchanges and cooperation.
2017	National action Plan on rational use of antibacterial in children (2017–2020)	NHFPC	Regulations on clinical use of antibacterial in children, surveillance of antimicrobial resistance, public education, and training of healthcare workers.
2017	A notice on further strengthening the clinical use of antibacterial and curbing bacterial resistance	NHFPC	To propose some requirements for weak links in the clinical use of antimicrobials
2018	A notice on continuous improving clinical use of antibacterial	NHC	To accelerate the construction of multidisciplinary teams of antibacterial management, diagnosis and treatment, strengthen the clinical use of antimicrobials in vulnerable populations, such as children.
2018–2021	Surveillance Plan for Antibiotic resistant zoonotic bacteria (2018, 2019, 2021)	MoA	To strengthen the monitoring of antibiotic resistance in animal sources, promote the rational use of medicines, and ensure the safety of animal source foods.
2020	A notice on improving surveillance on clinical use of antibacterial and bacterial resistance in children	NHC	A total of 67 Children’s hospitals or Maternal and Child Health Hospitals across the country were selected as the surveillance sites.

MoA: Ministry of Agriculture; NHFPC: National Health and Family Planning Commission (former name of National Health Commission); NHC: National Health Commission.
